# A machine learning algorithm predicts molecular subtypes in pancreatic ductal adenocarcinoma with differential response to gemcitabine-based versus FOLFIRINOX chemotherapy

**DOI:** 10.1371/journal.pone.0218642

**Published:** 2019-10-02

**Authors:** Georgios Kaissis, Sebastian Ziegelmayer, Fabian Lohöfer, Katja Steiger, Hana Algül, Alexander Muckenhuber, Hsi-Yu Yen, Ernst Rummeny, Helmut Friess, Roland Schmid, Wilko Weichert, Jens T. Siveke, Rickmer Braren

**Affiliations:** 1 Department of Diagnostic and Interventional Radiology, School of Medicine, Technical University of Munich, Munich, Germany; 2 Department of Pathology, School of Medicine, Technical University of Munich, Munich, Germany; 3 Department of Internal Medicine II, School of Medicine, Technical University of Munich, Munich, Germany; 4 Department of Surgery, School of Medicine, Technical University of Munich, Munich, Germany; 5 Division of Solid Tumor Translational Oncology, West German Cancer Center, University Hospital Essen, Essen, Germany; 6 German Cancer Consortium (DKTK) and German Cancer Research Center (DKFZ), Heidelberg, Germany; Centro Nacional de Investigaciones Oncologicas, SPAIN

## Abstract

**Purpose:**

Development of a supervised machine-learning model capable of predicting clinically relevant molecular subtypes of pancreatic ductal adenocarcinoma (PDAC) from diffusion-weighted-imaging-derived radiomic features.

**Methods:**

The retrospective observational study assessed 55 surgical PDAC patients. Molecular subtypes were defined by immunohistochemical staining of KRT81. Tumors were manually segmented and 1606 radiomic features were extracted with *PyRadiomics*. A gradient-boosted-tree algorithm was trained on 70% of the patients (N = 28) and tested on 30% (N = 17) to predict KRT81+ vs. KRT81- tumor subtypes. A gradient-boosted survival regression model was fit to the disease-free and overall survival data. Chemotherapy response and survival were assessed stratified by subtype and radiomic signature. Radiomic feature importance was ranked.

**Results:**

The mean±STDEV sensitivity, specificity and ROC-AUC were 0.90±0.07, 0.92±0.11, and 0.93±0.07, respectively. The mean±STDEV concordance indices between the disease-free and overall survival predicted by the model based on the radiomic parameters and actual patient survival were 0.76±0.05 and 0.71±0.06, respectively. Patients with a KRT81+ subtype experienced significantly diminished median overall survival compared to KRT81- patients (7.0 vs. 22.6 months, HR 4.03, log-rank-test P = <0.001) and a significantly improved response to gemcitabine-based chemotherapy over FOLFIRINOX (10.14 vs. 3.8 months median overall survival, HR 2.33, P = 0.037) compared to KRT81- patients, who responded significantly better to FOLFIRINOX over gemcitabine-based treatment (30.8 vs. 13.4 months median overall survival, HR 2.41, P = 0.027). Entropy was ranked as the most important radiomic feature.

**Conclusions:**

The machine-learning based analysis of radiomic features enables the prediction of subtypes of PDAC, which are highly relevant for disease-free and overall patient survival and response to chemotherapy.

## Introduction

Pancreatic ductal adenocarcinoma (PDAC) carries the worst prognosis of all tumor entities. Complete resection, often combined with an adjuvant chemotherapy regimen, remains the only curative therapy option in PDAC. In the metastatic setting, gemcitabine/nab-paclitaxel or FOLFIRINOX-based chemotherapy have been the mainstay in the treatment of PDAC [1–3]. However, although both intensified treatment protocols increased response rates up to approximately 30%, a substantial number of patients does not respond or acquires resistance in a considerably short time. Pre-clinical and clinical evidence suggests differential response of specific PDAC subtypes to these treatments. Among these, a particularly aggressive subtype, termed quasi-mesenchymal, basal-like or cytokeratin 81 positive (KRT81+) [4,5] has been investigated and found to be more sensitive to gemcitabine treatment in vitro [6] and less sensitive to FOLFIRINOX in a prospective clinical trial [7]. Thus, pre-therapeutic identification of specific subtypes in pancreatic cancer is urgently required to guide individual treatment decision.

So far, molecular profiling has relied on tissue biopsies, which are prone to undersampling, not least because of this entity’s morphological heterogeneity, which manifests as a heterogenic mix of tumor cell clusters, stroma and non-tumoral cell infiltrates. In addition, molecular subtyping requires high tissue quality and is both costly and time consuming, thus at current not introduced in routine patient care.

Non-invasive diffusion weighted-magnetic resonance imaging (DW-MRI, DWI), is an imaging technique which is part of the routine diagnostic work-up in many centers. It measures the random motion of water molecules and can thus quantify tissue microstructure and heterogeneity with high sensitivity [8]. Radiomics, i.e. the computer-based analysis of non-perceptual image features, provides a novel tool for the evaluation of DWI beyond traditional descriptive radiology. Recent work has shown its potential in e.g. the differentiation of tumor grading or the prediction of therapy response and survival in various tumor entities including PDAC [9,10].

In the current study we developed a machine learning algorithm capable of predicting clinically relevant histopathological PDAC subtypes from pre-operative DW-MRI derived ADC maps, evaluated tumor subtype-stratified overall survival for different chemotherapy regimens and assessed the clinical utility of this radiomic algorithm in the prediction of patient survival and chemotherapy response.

## Materials and methods

### Study design

The study was designed as a retrospective observational cohort study matched on histopathological tumor subtype.

Data collection, processing and analysis were approved by the institutional ethics committee (Ethics Commission of the Faculty of Medicine of the Technical University of Munich, protocol number 180/17). The requirement for consent was waived. All procedures were carried out in accordance to pertinent laws and regulations.

The STROBE checklist and inclusion flowchart can be found in S1 File. In brief, we considered 102 consecutive patients with final histopathological diagnosis of PDAC of the head or body for inclusion in the study. Patients without a final diagnosis of PDAC, with *unclassifiable* tumor subtype, who had undergone prior therapy (chemotherapy, resection prior to enrolment), died within the first 6 weeks of follow-up (to limit bias from postoperative complications), did not undergo the full imaging protocol or did not have technically sufficient imaging available (due to e.g. motion artifacts or stent placement), were excluded. A total of 55 patients who underwent surgical resection in curative intention were included in the study using histopathological subtype as the matching criterion. 27 patients with a KRT81+ subtype and 28 patients with a KRT81- subtype [5] were included. The follow-up interval began on the 1^st^ of January, 2010 and ended on the 31^st^ of December 2016. All patients died within the follow-up interval thus observed (uncensored) endpoint data is available for all patients. For 21 patients, follow-up data and histopathological data was sourced from the “PR2” cohort described in [5]. For all other patients, clinical follow-up was handled by the departments of surgery and internal medicine, clinical data was sourced from the hospital’s clinical system and histopathological data was generated during the study. Radiomic data for all patients was generated during data analysis. All analyses were performed on pseudonymized datasets by separate individuals (G.K. and S.Z.) from January to May 2019.

### Clinical data

The following clinical data was collected: age at diagnosis, sex, pTNM, R, G, tumor volume (from the final histopathological report), ECOG-status, adjuvant chemotherapy (gemcitabine-based vs. no chemotherapy), palliative chemotherapy (gemcitabine-based vs. FOLFIRINOX) and lymph-node ratio (LNR). Disease-free survival was defined as the time from diagnosis to tumor recurrence or occurrence of metastatic disease and overall survival as the time from diagnosis to disease-related death.

### Imaging data

Patients underwent magnetic resonance imaging (MRI) at 1.5T (Siemens Magnetom Avanto, release VB17). The protocol included the following sequences: axial and coronal T2-weighted spin echo (SE) images at 5mm; axial T1w gradient echo (GE) images at 5mm before contrast media injection and during the arterial, pancreatic parenchymal, portal-venous, systemic venous and delayed phases (as determined by testing bolus injection); axial unidirectional diffusion-weighed imaging at b-values of 0, 50, 300 and 600 with echo-planar imaging (EPI) readout and ADC map calculation. ADC map reconstructions were 5.5x5.5x5 mm (xyz) to a 192x192 voxel matrix. Furthermore, single-shot T2w magnetic resonance cholangiopancreatography (MRCP) was performed and reconstructed as a radial maximum intensity projection (MIP) series. The imaging protocol, and the technical software and hardware specifications of the MRI machine remained unaltered during the data acquisition period.

### Image segmentation

The datasets were exported in pseudonymized form to a segmentation workstation running ITK-SNAP v. 3.8.0 (beta). Segmentation was performed under radiological reporting room conditions by consensus reading of two experienced observers (G.K. and S.Z.). After a period of two weeks, datasets were shuffled by a third person (F.L.) and segmented again by the same observers. Segmentations were then quality-controlled by an abdominal radiologist with >10 years of experience in pancreatic MRI (R.B) and the best segmentations retained. Segmentation was performed manually in the b = 600 images and transferred to the ADC maps. All other sequences were available to observers for anatomical correlation.

### Biostatistical and machine learning modeling

For assessing bias due to clinical confounders, overall survival time was evaluated by a multivariate *Cox proportional hazards* model. The distributions of covariates were compared between groups with different histopathological subtype using *Fisher’s exact test*.

Biostatistical modeling was performed using the Python (v.3.7.3) packages *Lifelines* and *Scikit-Survival* [11]. Kaplan-Meier-Plots were drawn in GraphPad Prism (v.8). For all inferential statistical procedures, a P-value of <0.05 was considered statistically significant.

Image postprocessing, feature extraction, feature preprocessing, feature engineering and machine learning modeling are described in S1 File. In brief, radiomic features were derived using *PyRadiomics* (v. 2.1) [12] yielding a total of 1606 features, of which 40 were retained after exclusion of features with low-variance or repeated segmentation instability. Unless otherwise noted, a randomized, 10-fold shuffle-splitting cross-validation strategy was used with 70% (N = 38) of the cohort used for training and 30% (N = 17) for testing.

For the prediction of tumor subtype, a supervised *Gradient Boosted Decision Tree* model (*XGBoost* [13], instantiated as a binary classifier within the Python library *scikit-learn*) was fit with histopathological subtype as a binary label to the radiomic features and tested for predictive sensitivity, specificity and ROC-AUC. Significance testing for model evaluation metrics was carried out using permutation testing [14]. The threshold probability for classification was the default value of .50. Feature importance was assessed by the inbuilt feature importance classifier (using the “gain” parameter).

For survival modeling, two strategies were followed: For assessing the agreement between model survival predictions and actual patient survival, the cohort was split into an interleaved training/testing set of 70%/30% and a stochastic gradient boosted survival regression model was trained using a subsampling tree building approach. The concordance indices between the resulting proportional hazards survival model and the actual patient survival of the unseen holdout set were calculated separately for disease-free and overall survival. 95% confidence intervals were calculated by bootstrap resampling. For evaluating the capacity of the model to separate between patients with a high and low survival risk, disease-free and overall survival were assessed for patients for whom the algorithm predicted a KRT81+ subtype (designated as KRT81+ or *high risk* signature) vs. patients predicted KRT81- (*low risk* signature). The Kaplan-Meier/ log-rank methods were used to compare the survival functions in the cross-validation folds.

Finally, for the assessment of chemotherapy sensitivity, disease-free and overall survival were evaluated stratified by radiomic signature (KRT81+/*high risk* vs. KRT81-/*low risk*) and chemotherapy regimen under the assumption of a differential response of the subtypes identified by the signature to the applied chemotherapy regimen, using the Kaplan-Meier/log-rank methods in the cross-validation folds.

### Histopathological workup

**Fig 1:**
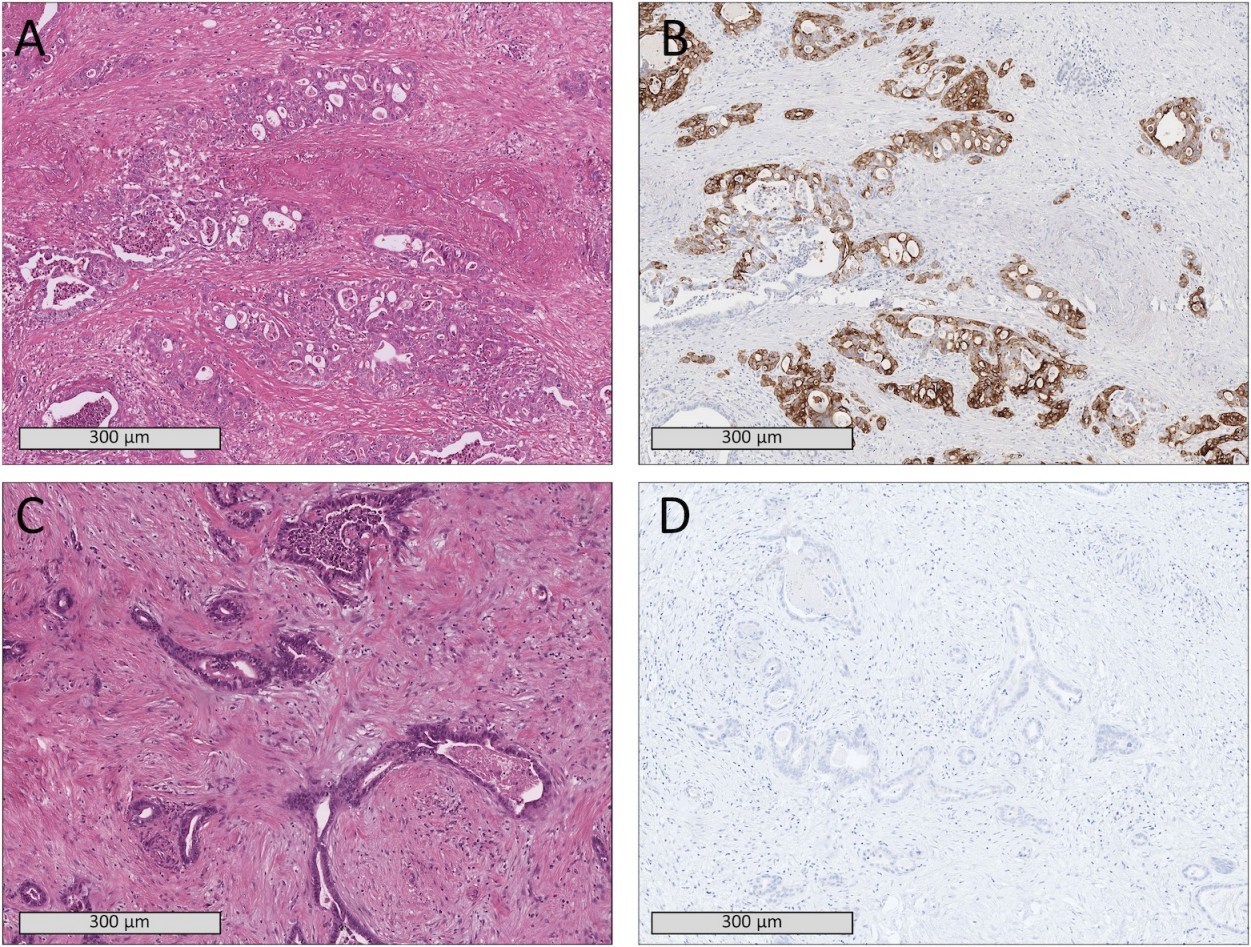
Histopathological samples of two patients showing comparable tissue morphology in H&E staining (A,C) but a KRT81+ subtype (B) in one patient and KRT81- subtype (D) in the other patient.

Histopathological staining and immunohistochemical workup were performed by application of surrogate markers to determine the molecular subtype of PDAC based on the previously established immunohistochemical protocol described in [5]. In brief, 2μm sections were stained for HNF1a and KRT81+ and tumors were categorized into either one of two classes: KRT81+[/HNF1a-] or KRT81-[/HNF1a+](Fig 1). Tumors positive or negative for both markers were excluded, the former due to recently reported suspicions of contamination with acinar cells [15], the latter due to unclassifiability.

## Results

**Fig 2:**
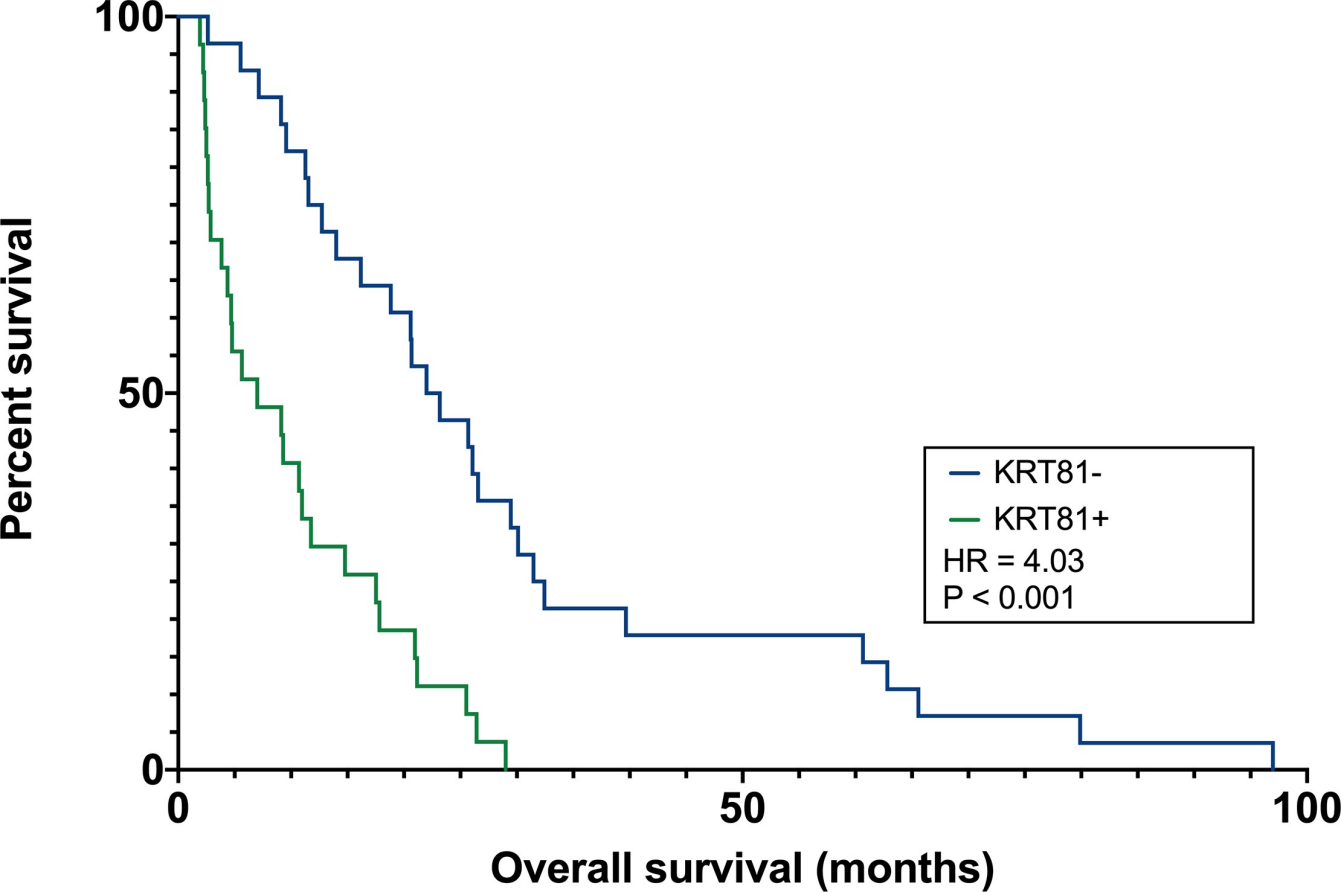
Patients with a KRT81+ subtype experienced significantly diminished overall survival.

The molecular subtype of PDAC was significantly associated with overall survival. Patients with a KRT81+ subtype experienced significantly diminished overall survival (7.0 [1.93 to 29.0] vs. 22.6 [2.63 to 96.97] months median survival, HR 4.03 log-rank-test P = <0.001, Fig 2, Table 1). No other covariate was significantly associated with overall survival in this cohort and the baseline distribution of clinical covariates did not differ significantly between the two patient subcohorts (Table 2).

**Table 1:** Multivariate Cox proportional hazards analysis results of clinical parameters.

*Parameter*	coef	exp(coef)	p	lower 0.95	upper 0.95
***Subtype*** ***(KRT 81+ vs. KRT 81-)***	1.44	4.03	<0.001	0.76	2.12
***pN (0 vs. 1)***	1.20	3.32	0.20	-0.63	3.03
***Age***	0.02	1.02	0.30	-0.01	0.05
***pM (0 vs. 1)***	0.50	1.65	0.30	-0.45	1.44
***Palliative CTX*** ***(Gem mono vs. FOLFIRINOX)***	-0.39	0.68	0.32	-1.16	0.38
***pT***	0.22	1.25	0.36	-0.25	0.70
***Tumor Volume***	-0.01	0.99	0.38	-0.04	0.01
***Grading (2 vs. 3)***	0.21	1.24	0.49	-0.40	0.83
***Adjuvant CTX*** ***(Gem-based vs. None)***	-0.45	0.64	0.53	-1.85	0.95
***LNR***	-2.69	0.07	0.65	-14.23	8.85
***R (0 vs. 1)***	-0.14	0.87	0.69	-0.84	0.56
***Sex (F vs. M)***	0.08	1.08	0.81	-0.57	0.73
***ECOG (0 vs. 1)***	-0.06	0.94	0.88	-0.81	0,70

**Table 2:** Distribution of clinical parameters between the cohorts with KRT81+ and KRT81- tumor subtypes alongside crosstabulation results.

Parameter	KRT 81+ Subcohort (27)	STDEV	KRT 81- Subcohort (28)	STDEV	P
**Age**	67	11.7	65	10.5	.52
**Adjuvant CTX**	Gem-based: 25,Did not receive: 2		Gem-based: 26, Did not receive: 2		-
**Palliative CTX**	Gem-based: 14, FOLFIRINOX: 13		Gem-based: 16, FOLFIRINOX: 12		.78
**Experienced Event**	Yes: 27		Yes: 28		-
**G**	2: 16, 3: 11		2: 15, 3: 13		.79
**pM**	0: 22,1: 5		0: 23,1: 5		1.0
**pN**	0: 6, 1: 21		0: 8, 1: 20		.76
**pT**	1: 3, 2: 2, 3: 22		1: 3, 2: 3, 3: 22		-
**R**	0: 20, 1: 7		0: 21, 1: 7		1.0
**Sex**	Female: 12, Male: 15		Female: 13,Male: 15		1.0
**ECOG**	0:11,1:16		0:13,1:15		.79
**Tumor Volume (ml)**	16.4	15.6	15.0	14.0	.72
**Lymph Node Ratio**	0.12	0.07	0.10	0.07	.29

P: Fisher’s exact test P

**Fig 3:**
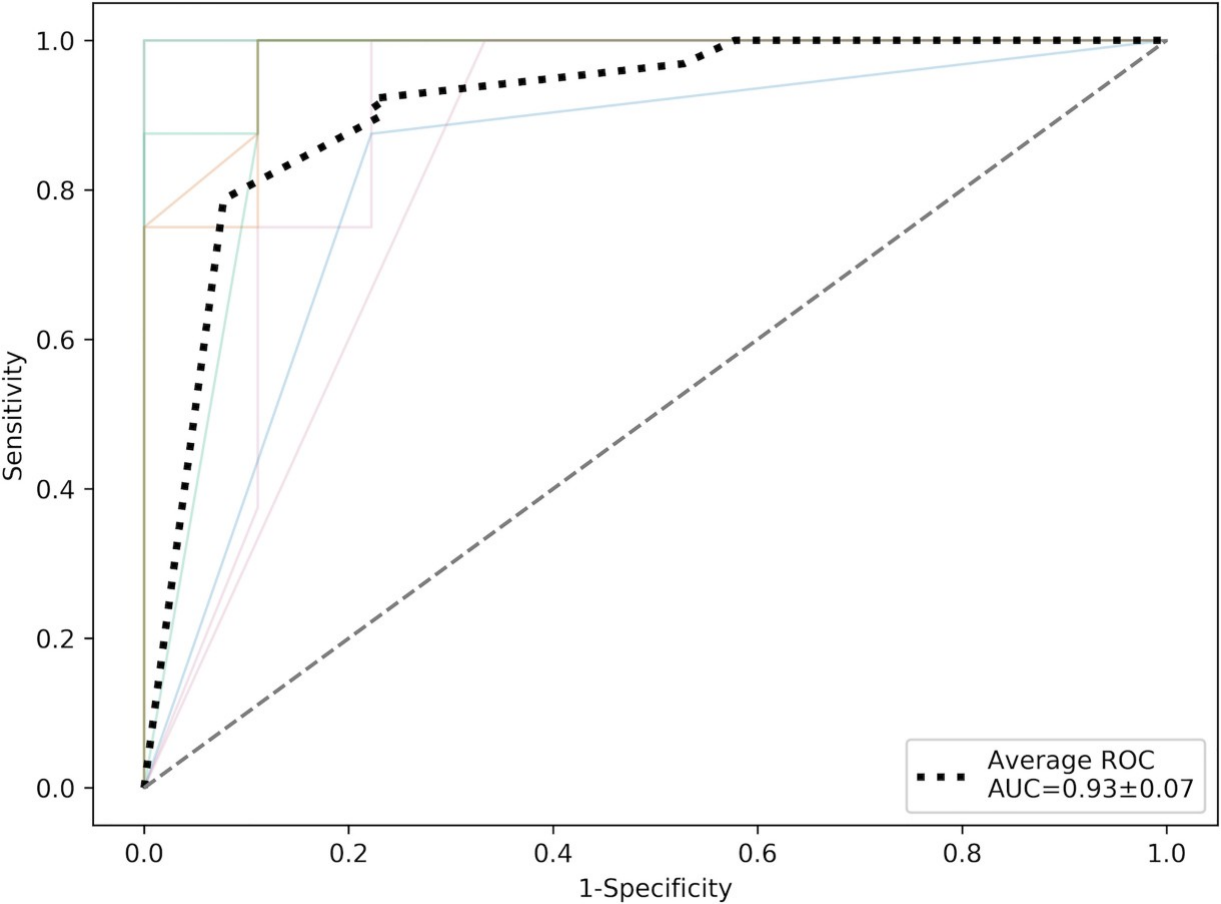
ROC curves (colored) and average ROC-curve (black dotted) over 10 random stratified shuffle-splits of the dataset.

The machine learning algorithm achieved a mean±STDEV sensitivity, specificity and ROC-AUC of 0.90±0.07, 0.92±0.11, and 0.93±0.07, respectively; all P = 0.01 (Fig 3).

The feature importance evaluation of the algorithm yielded 13 radiomic parameters with an importance greater than zero for the classification process. Among these, entropy, a radiomic feature derived from the histogram of the original image and signifying the degree of heterogeneity in the tumor region [16], was classified as the most important feature by a large margin. All features alongside their importance metrics can be found in Table 3 and further information about the radiomic parameters can be found in S1 File.

**Table 3:** Radiomic features alongside their importance as ranked by the algorithm.

Feature	Importance
**original_firstorder_Entropy**	0.73
**gradient_firstorder_Kurtosis**	0.10
**log-sigma-1-0-mm-3D_glcm_Imc2**	0.09
**log-sigma-3-0-mm-3D_firstorder_Kurtosis**	0.05
**original_glszm_SizeZoneNonUniformityNormalized**	0.005
**wavelet-HHL_glcm_Imc2**	0.005
**wavelet-HHL_glszm_SmallAreaEmphasis**	0.004
**wavelet-HHL_glszm_ZonePercentage**	0.003
**original_shape_Maximum2DDiameterRow**	0.003
**log-sigma-2-0-mm-3D_glszm_SmallAreaHighGrayLevelEmphasis**	0.002
**original_glszm_LargeAreaLowGrayLevelEmphasis**	0.001
**wavelet-HLL_glszm_ZonePercentage**	0.001
**wavelet-LHL_firstorder_Kurtosis**	0.0005

To test the association of the radiomic parameters with patient survival, a survival regression model including all radiomic features was developed. The concordance index between model predictions and actual survival on the entire dataset was 0.76±0.05 [95% CI 0.66–0.86] for disease-free and 0.71±0.06 [95% CI 0.60–0.80] for overall survival.

**Fig 4:**
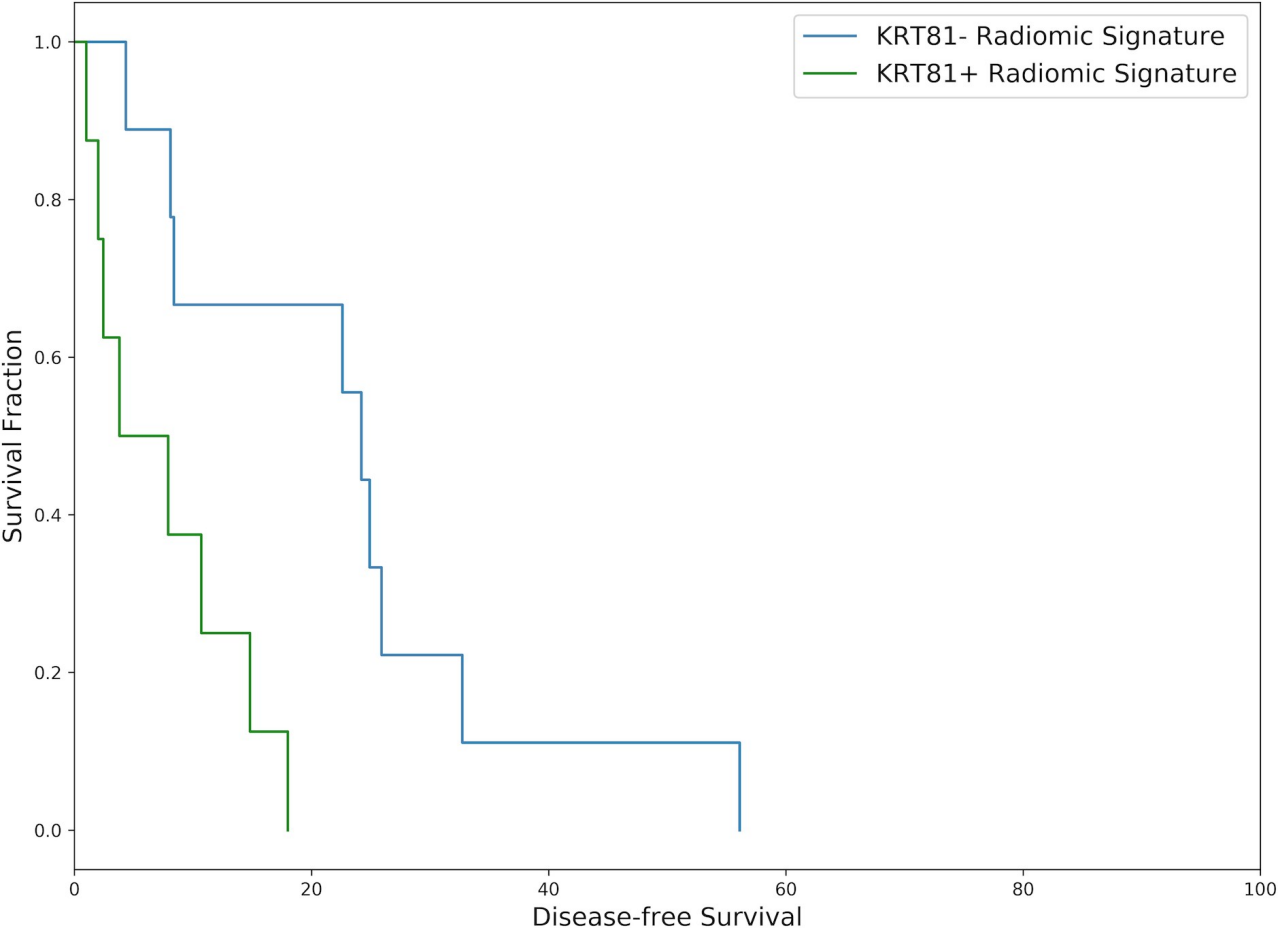
Exemplary Kaplan-Meier disease-free survival curve drawn from the 10 cross-validation folds representing the median hazard ratio. Patients with a *high risk* radiomic phenotype experienced significantly diminished survival (7.90 vs. 24.20 months median DFS, *log-rank-test* p = 0.004, HR = 3.17, N = 17).

**Fig 5:**
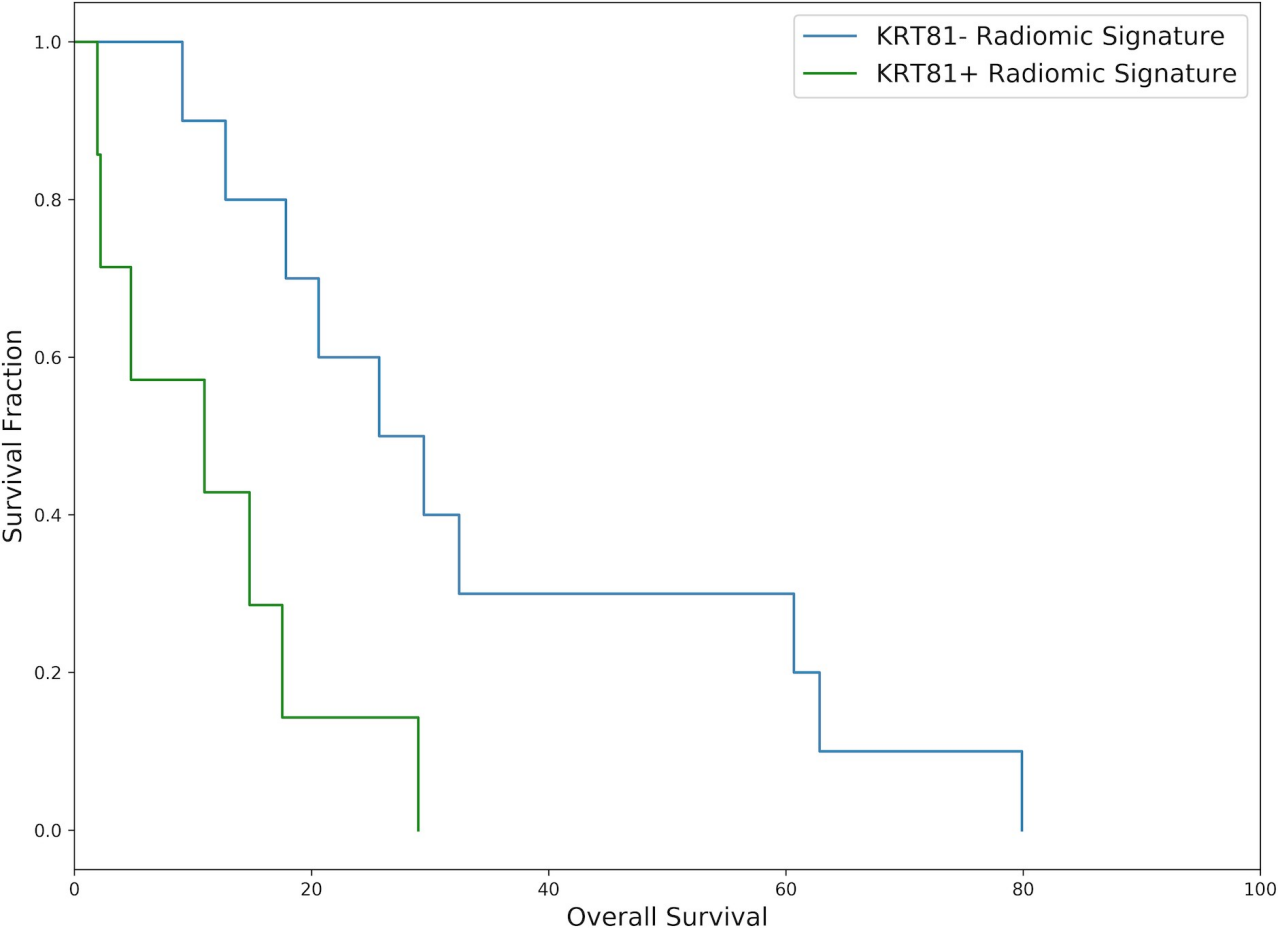
Exemplary Kaplan-Meier overall survival curve drawn from the 10 cross-validation folds representing the median hazard ratio. Patients with a *high risk* radiomic phenotype experienced significantly diminished survival (10.97 vs. 25.70 months median OS, *log-rank-test* p = 0.006, HR = 3.03, N = 17).

Patients with a KRT81+ (*high risk*) radiomic signature experienced diminished disease-free and overall survival. Over the 10 cross-validation folds, the median hazard ratio for patients with a *high risk* vs. a *low risk* radiomic signature was 3.08 [range 0.46 to 3.37] for disease-free and 3.04 [range 0.27 to 3.36] for overall survival. The radiomics-derived stratification led to a statistically significant difference in survival functions (*log-rank-test* p<0.05) in 7 out of 10 cross-validation folds for disease-free and in 8 out of 10 folds for overall survival. Figs 4 and 5 show exemplary survival curves. All survival curves can be found in the S1 File.

**Fig 6:**
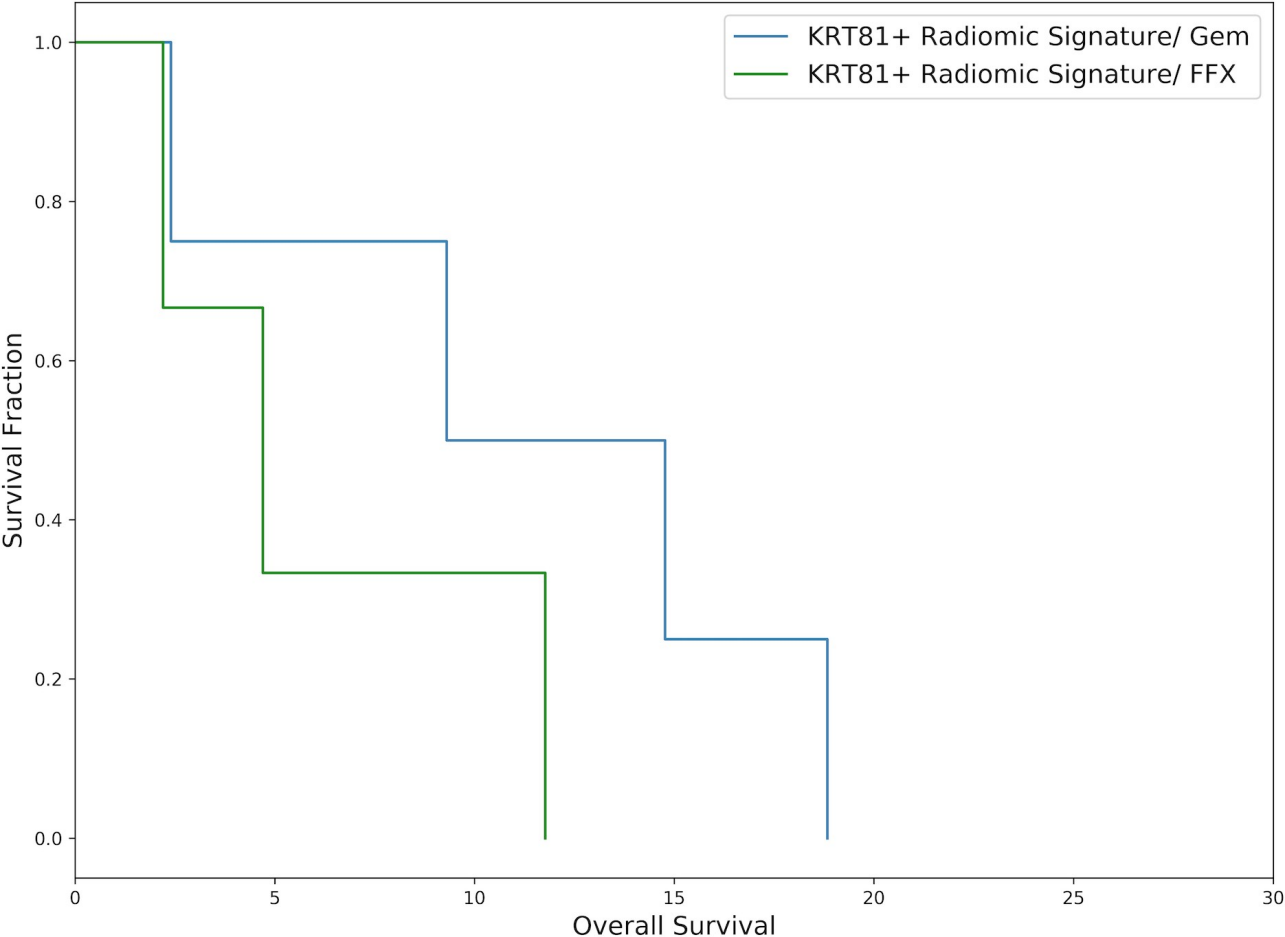
Exemplary Kaplan-Meier plot of patients with a predicted KRT81+/ *high risk* radiomic signature drawn from the 10 cross-validation folds and representing the median hazard ratio. Patients who received gemcitabine experienced improved survival, although no statistical significance is observed in this case (median survival 4.70 vs 9.30 months, p = 0.23, HR = 1.03, N = 7).

**Fig 7:**
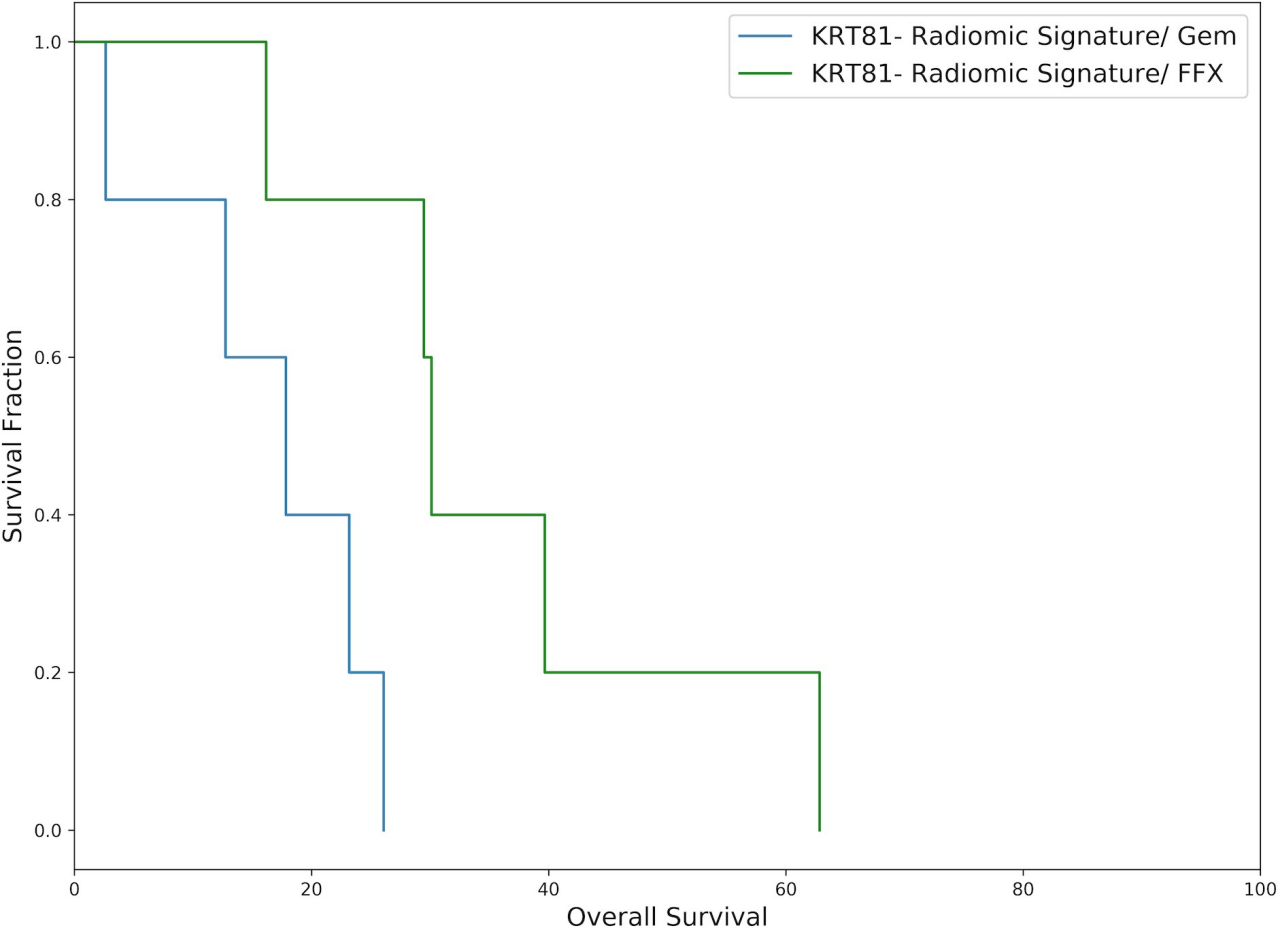
Exemplary Kaplan-Meier plot of patients with a predicted KRT81-/ *low risk* radiomic signature drawn from the 10 cross-validation folds and representing the median hazard ratio. Patients who received FOLFIRINOX experienced significantly improved survival, (median survival 17.83 vs 30.10 months, p = 0.01, HR = 2.57, N = 10).

For evaluating the potential of the radiomic algorithm to inform the choice of palliative chemotherapy regimen, overall survival was evaluated stratified by radiomic signature (KRT81+/*high risk* vs. KRT81-/*low risk*) and assessed separately by selected chemotherapy regimen. Patients with a KRT81+ radiomic signature experienced prolonged overall survival under palliative gemcitabine therapy compared to FOLFIRINOX (median HR 1.13 [range 0.03 to 2.57]) but statistical significance was only observed for *log-rank-tests* of 2 out of 10 cross-validation folds. Inversely, patients with a KRT81- radiomic signature experienced improved overall survival under palliative FOLFIRINOX compared to gemcitabine (median HR 2.89 [range 0.99 to 3.34] with statistical significance observed in 6 out of 10 cross-validation folds. Figs 6 and 7 show exemplary survival curves and all survival curves can be found in the S1 File.

**Fig 8:**
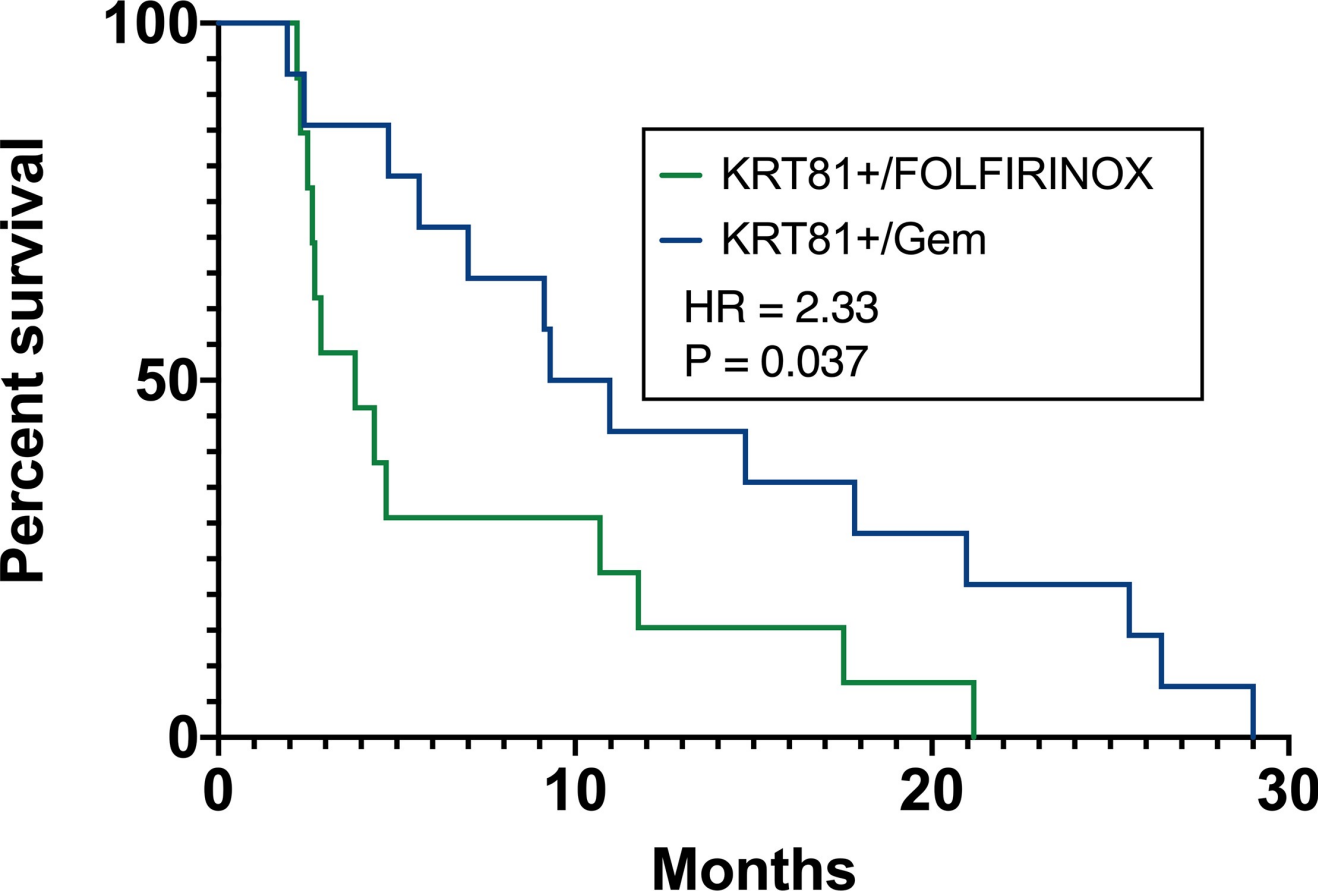
Patients with KRT81+ subtype experience longer overall survival under palliative gemcitabine chemotherapy.

**Fig 9:**
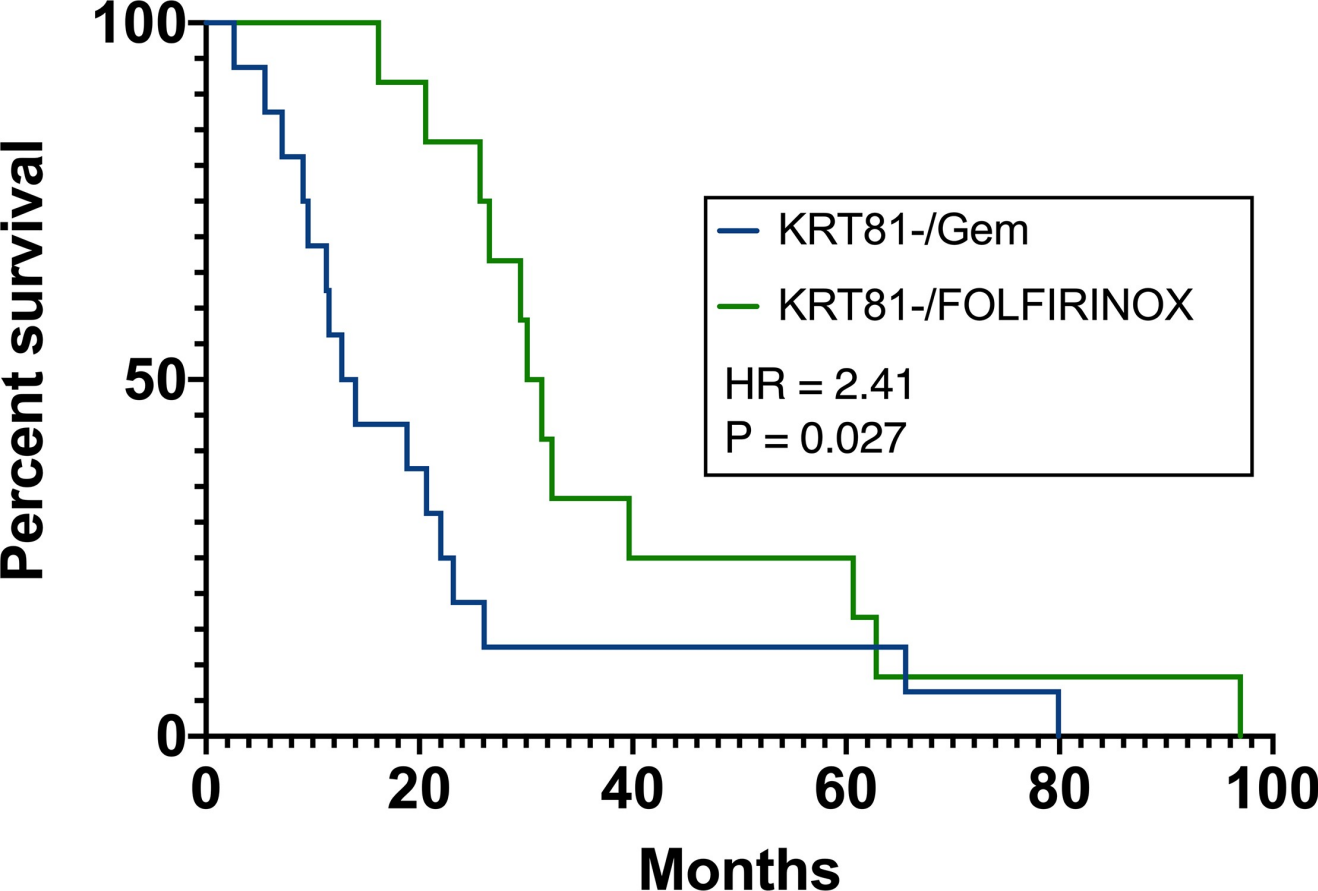
Patients with KRT81- subtype experience improved survival under palliative FOLFIRINOX chemotherapy.

Lastly, overall survival was evaluated separately for histopathological subtypes stratified by chemotherapy regimen. Patients with a KRT81+ histopathological subtype who received gemcitabine-based palliative chemotherapy experienced significantly improved survival compared to patients with KRT81+ tumors who received FOLFIRINOX (10.14 vs. 3.8 months median survival, HR 2.33, P = 0.037, Fig 8). Conversely, KRT81- subtype patients experienced significantly improved survival under FOLFIRINOX chemotherapy compared to gemcitabine-based regimens (30.8 vs. 13.4 months median survival, HR 2.41, P = 0.027, Fig 9).

## Discussion

In this exploratory study, we demonstrate that radiomic analysis of ADC maps paired with machine-learning modeling can discriminate with high sensitivity and specificity between two groups of histomorphologically defined molecular subtypes of pancreatic ductal adenocarcinoma (PDAC), associated with significantly different disease-free and overall survival and predictive of distinct responses to commonly employed chemotherapeutic regimens in the palliative setting. Although our findings should be interpreted mindful of the small cohort size, we provide evidence for the utility of radiomics and machine learning for the non-invasive therapy stratification and survival risk-assessment of pancreatic cancer patients.

The potential of non-invasive imaging-derived biomarkers (from non-perceptual image features or source data) has been demonstrated in several studies with the prediction of tumor genetics and patient outcome [17–19]. However, their widespread application beyond proof-of-principle studies requires the identification of stable and reproducible parameters, embedded within a standardized and quality-controlled workflow [20–23].

Among the parameters tested for classification in our study, Entropy was ranked the most important by the algorithm. Entropy and Entropy-related features, which express disorder and heterogeneity of the image and -by extension- are hypothesized to mirror tumoral heterogeneity itself, have been demonstrated in (meta-)analyses of several different tumor entities and across imaging modalities as reliable and repeatable quantitative parameters [24–26].

Considering the sampling errors immanent in this histopathologically heterogeneous tumor entity, the complexity of mutational events (e.g. variable amounts of mutational *Kras* [27] and the likelihood of ongoing transitional processes, Entropy as a continuous variable can be imagined as a non-invasive measure of the KRT81+ partition of the tumor region. To test this hypothesis would require an integrated whole-tumor analysis, including high resolution, data-rich imaging, histopathology and molecular profiling [28].

The rapid evolution of new therapeutic options in the treatment of PDAC requires the development of markers for a reliable pre-therapeutic patient stratification and -in light of the above-mentioned tumoral plasticity, therapy monitoring. Conroy et al. demonstrated significantly improved survival rates of FOLFIRINOX over Gemcitabine monotherapy in the palliative setting [3]. However, the COMPASS trial [7] demonstrated differential response of the basal-like versus non-basal-like PDAC subtypes to FOLFIRINOX treatment, which is well in accordance with our study results. If further validated in prospective trials, these findings could have tremendous implications in patient stratification and subtype-guided therapy selection. In addition, targeted therapies such as Olaparib, are highly effective yet even more specific for a certain molecular profile [29] and many new targeted, stroma- and immune-based treatment strategies are being explored. This increasing complexity requires robust and cost-efficient tools for clinically relevant patient stratification to best leverage current knowledge and advance the field. Informed decision based on molecular profiling (microdissection and genome sequencing) as applied in the COMPASS trial faces serious limitations (i.e. sampling error, high cost) and is therefore currently not feasible in routine patient care. Quantitative noninvasive imaging, and especially radiation and contrast-free quantitative modalities such as DWI may serve this purpose and are thus excellent candidates for exploration in a prospective trial design.

Limitations of this study are the small cohort size, precluding statistical significance in the survival predictions and necessitating a cross-validation approach, and the lack of an external testing cohort as well as the retrospective, single-center nature of the investigation. Such issues are still common in the imaging field and compounded by the lack of standardization in sequence acquisition between institutions and of overarching registers or study centers permitting patient pooling. Recently, initiatives have arisen to combat some of these issues by harmonization of MRI protocols [30] and the standardization of imaging markers [31].

In conclusion, our study is an exploratory venture into the field of quantitative imaging analysis and radiology/pathology-correlation in PDAC. We encourage the validation of our findings in a larger cohort and in a prospective trial design.

## Supporting information

S1 File (PDF)
