# Supplementary material for: A machine learning algorithm predicts molecular subtypes in pancreatic ductal adenocarcinoma with differential response to gemcitabine-based versus FOLFIRINOX chemotherapy
Source: PLoS One. 2019 Oct 2;14(10):e0218642. doi: 10.1371/journal.pone.0218642 (PMC6774515; doi:10.1371/journal.pone.0218642)
Supplement: S1 File — (PDF) [file pone.0218642.s001.pdf]

A machine learning algorithm predicts molecular subtypes in pancreatic ductal adenocarcinoma with differential response to gemcitabine-based versus FOLFIRINOX chemotherapy

Georgios Kaissis[1¶], Sebastian Ziegelmayer[1¶], Fabian Lohöfer[1¶], Katja Steiger[2], Hana Algül[3], Alexander Muckenhuber[2], Hsi-Yu Yen[2], Ernst Rummeny[1], Wilko Weichert[2], Roland Schmid[3], Helmut Friess[4], Jens T. Siveke[5,6] and Rickmer Braren[1*]

[1]Department of diagnostic and interventional radiology, School of Medicine, Technical University of Munich, Munich, Germany;
[2]Department of Pathology, School of Medicine, Technical University of Munich, Munich, Germany;
[3]Department of Internal Medicine II, School of Medicine, Technical University of Munich, Munich, Germany;
[4]Department of Surgery, School of Medicine, Technical University of Munich, Munich, Germany;
[5]Division of Solid Tumor Translational Oncology, West German Cancer Center, University Hospital Essen, Essen, Germany.
[6]German Cancer Consortium (DKTK) and German Cancer Research Center (DKFZ), Heidelberg, Germany

Supplemental Material

1 Feature extraction

Tumors were manually segmented by consensus reading as detailed in the main manuscript. Radiomic features were extracted from the quality-controlled 3-dimensional volumes as follows: Intensity discretization was performed to a fixed bin number of 32 bins for all scans as described in (1). No normalization was performed. Value plausibility was ascertained for all parameters. Images were spatially resampled to 3x3x3mm using the *BSpline* interpolator. No resegmentation was performed. Radiomic features were derived using PyRadiomics v. 2.1.1 (2) yielding 19 first order statistics, 16 3D shape-based, 10 2D shape based, 24 Gray Level Cooccurence Matrix, 16 Gray Level Run Length Matrix, 16 Gray Level Size Zone Matrix, 5 Neighbouring Gray Tone Difference Matrix and 14 Gray Level Dependence Matrix features as well as Laplacian of Gaussian-filtered, wavelet-decomposition-based (using the *coiflet 1* function), square, exponential, gradient, square-root, logarithm and local binary-pattern filtered versions of these features. Laplacian of Gaussian-filtering was performed with a kernel (*sigma)* of 3mm. GLCM and GLRLM were extracted using the default settings (separately for each direction then averaged). Feature descriptions can be found in the PyRadiomics documentation (3). Feature extraction and processing was carried out according to the IBSI manual v. 7 (4). 1606 features were extracted in total.

2 Feature preprocessing

The following radiomic features were excluded: Features yielding nil, constant or-missing values. Furthermore, tumors were segmented a second time after 2 weeks by the same observers to test for repeated segmentation stability. The intra-class-correlation coefficient (two-way mixed effects model/ consistency as described by McGraw and Wong (5)) was calculated and features yielding inter-segmentation values below 0.8 were excluded. In total, the following 40 reproducible and stable features remained:

original_firstorder_Entropy
original_GLCM_Joint_Energy
wavelet-HLL_gldm_SmallDependenceEmphasis
wavelet-LHL_firstorder_Kurtosis
lbp-3D-k_glcm_Contrast
log-sigma-1-0-mm-3D_glcm_Imc2
wavelet-HHL_glszm_SmallAreaEmphasis
lbp-3D-k_firstorder_Variance
original_glszm_LargeAreaLowGrayLevelEmphasis
wavelet-HHL_glszm_ZonePercentage
log-sigma-1-0-mm-3D_glcm_Imc1
wavelet-HHL_glcm_Imc2
lbp-3D-k_firstorder_90Percentile
log-sigma-3-0-mm-3D_gldm_DependenceVariance
wavelet-LHH_firstorder_Kurtosis
wavelet-LHH_glcm_Imc2
lbp-3D-m2_firstorder_Kurtosis
wavelet-LHH_gldm_HighGrayLevelEmphasis
wavelet-HHL_glcm_DifferenceVariance
lbp-3D-k_glcm_Correlation
log-sigma-3-0-mm-3D_glszm_SmallAreaLowGrayLevelEmphasis
wavelet-HHL_glcm_SumSquares
log-sigma-3-0-mm-3D_firstorder_Kurtosis
wavelet-HLL_glszm_ZonePercentage
log-sigma-2-0-mm-3D_glszm_SizeZoneNonUniformity
log-sigma-3-0-mm-3D_gldm_LargeDependenceEmphasis
original_glszm_SizeZoneNonUniformityNormalized
original_gldm_DependenceVariance
original_glszm_LargeAreaEmphasis
wavelet-HHL_glszm_SmallAreaLowGrayLevelEmphasis
wavelet-LHL_glszm_SmallAreaEmphasis
log-sigma-2-0-mm-3D_glszm_SmallAreaHighGrayLevelEmphasis
log-sigma-1-0-mm-3D_glcm_ClusterProminence
original_shape_Maximum2DDiameterRow
wavelet-LLL_firstorder_Entropy
wavelet-LHH_glrlm_ShortRunHighGrayLevelEmphasis
gradient_firstorder_Kurtosis
log-sigma-2-0-mm-3D_firstorder_Median
log-sigma-2-0-mm-3D_glcm_Imc1
original_firstorder_Kurtosis

Potential intercorrelation between the relevant features selected by the algorithm was explored by calculating Spearman's *rho* between their values. The correlation between the features was found to be weak (as defined by Dancey and Reidy (6)) at best, signifying feature independence. A heatmap of feature correlation is seen below:

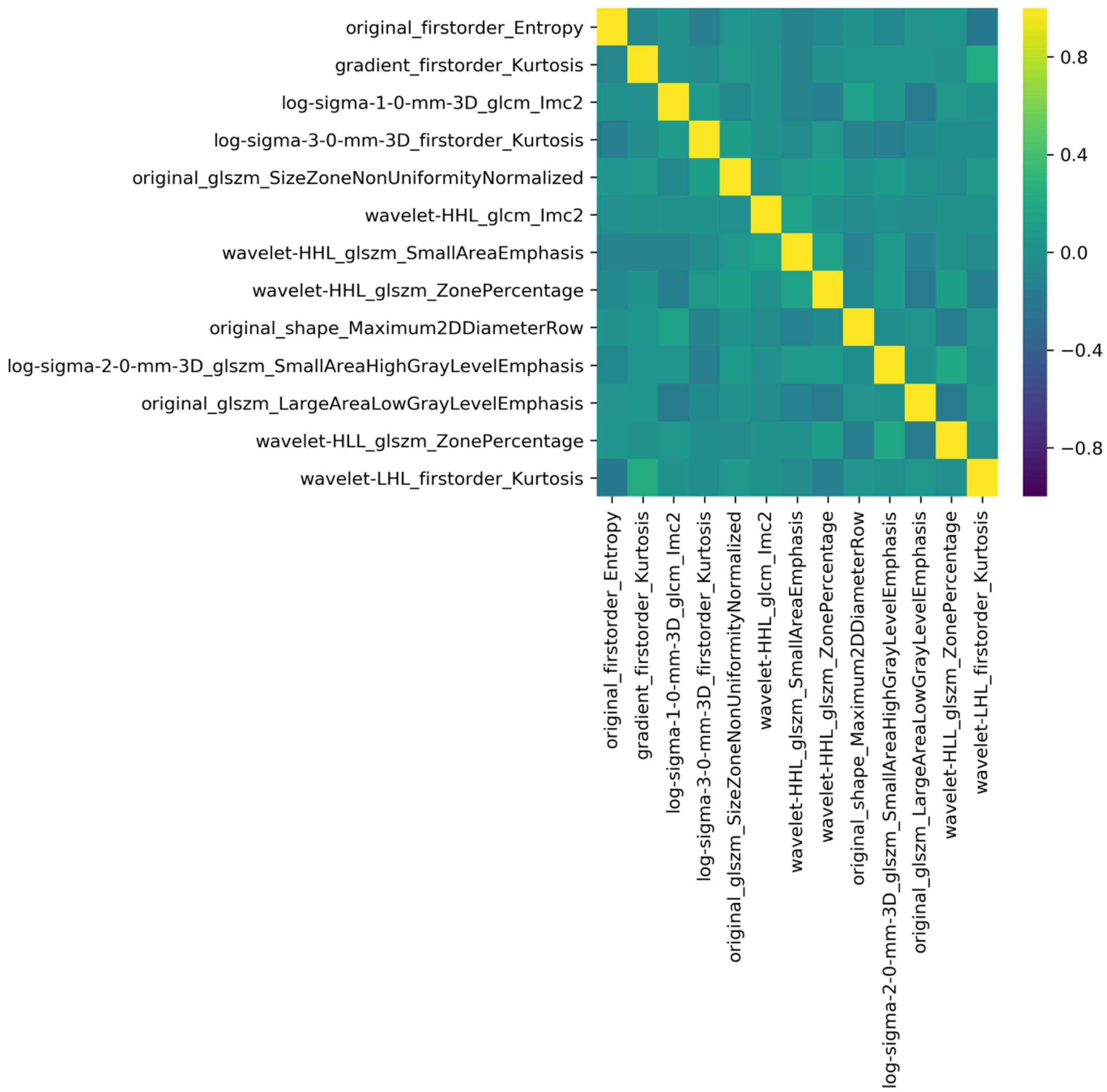

3 Feature engineering and machine learning modeling

Machine learning modeling was performed using the Python programming language version 3.7. The following packages were used: Pandas 0.23.4, Numpy 1.16.2, Matplotlib 3.0.3, Seaborn 0.9.0, Scikit Learn 0.20.3, Lifelines 0.21.0, Statsmodels 0.9.0, XGBoost 0.90., Scikit-Survival 0.9.

Feature values were normalized to a range of (0,1) using the StandardScaler package implemented in sklearn.preprocessing.

Because of the small dataset size, the use of automatic hyperparameter tuning and nested cross-validation was eschewed. Some manual hyperparameter modifications were made to reduce model variance and increase overfitting resistance: tree complexity penalization was

applied to the leaf values in the form of L2 regularization and shrinkage regularization was introduced by reducing the learning rate.

For training and testing, the estimator was fit and tested using stratified shuffle/split cross-validation with 10 splits of 70%/30% (train/test) of the dataset. For testing survival prediction, a manual train/test set was built using interleaving to preserve the time-series structure and a stochastic Gradient Boosted Regression Tree algorithm was used. The learning rate was set at 0.01 and individual trees were built using 90% of the rows. Cox proportional hazards was used as a loss function to optimize and trees were split based on the Friedman MSE criterion. The concordance index between the predictions of the model on the testing set and the actual survival times was calculated.

For all other survival analyses, the stratified shuffle/split cross-validation technique was used. The survival curves stratified by radiomic signature only and by both, radiomic signature and chemotherapy can be found below:

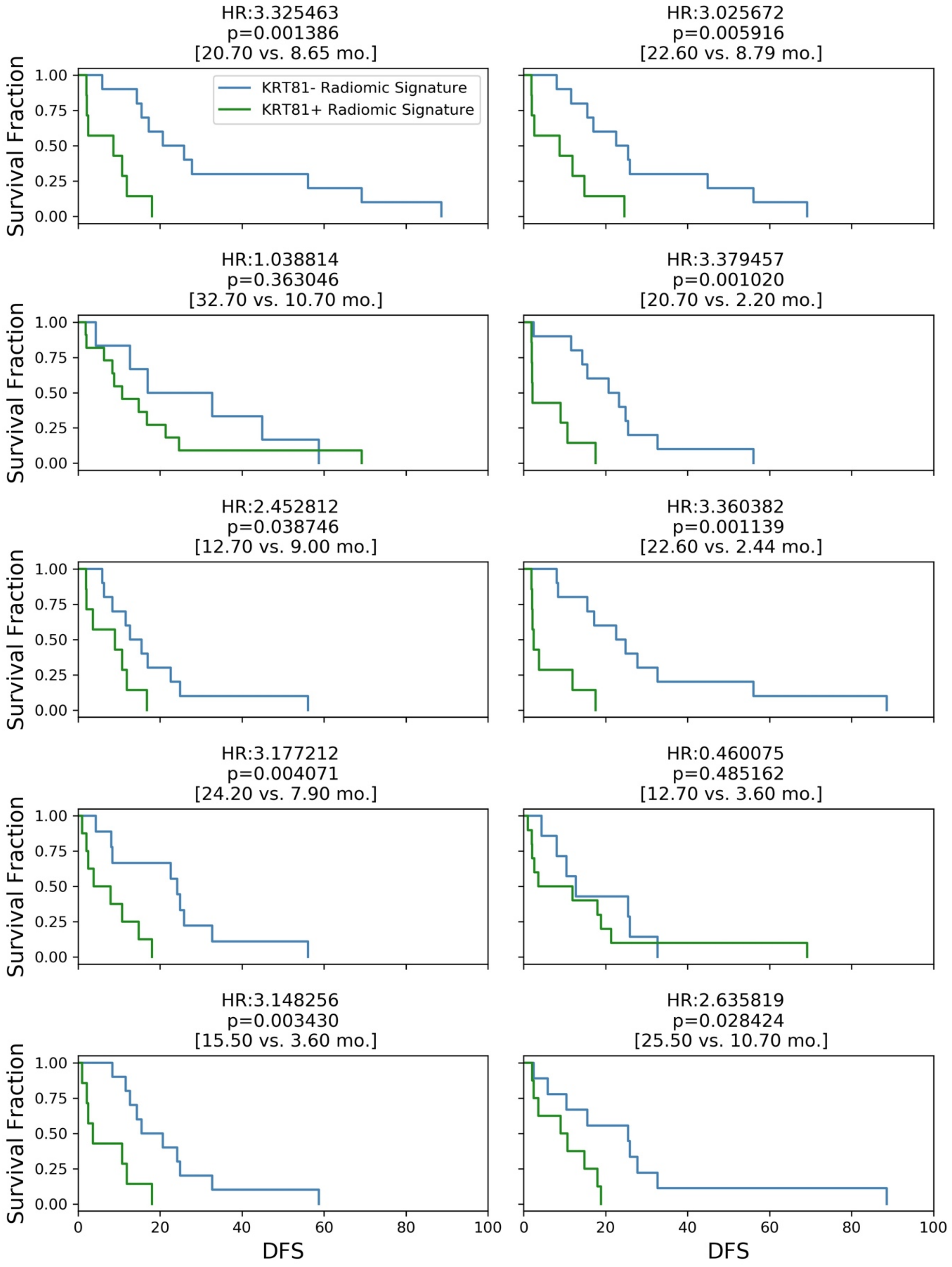

Kaplan-Meier curves resulting from 10-fold cross validation and showing disease-free survival stratified by radiomic signature (KRT81+/*high risk* (blue line) or KRT81-/*low risk* (green line)). Total N=17.

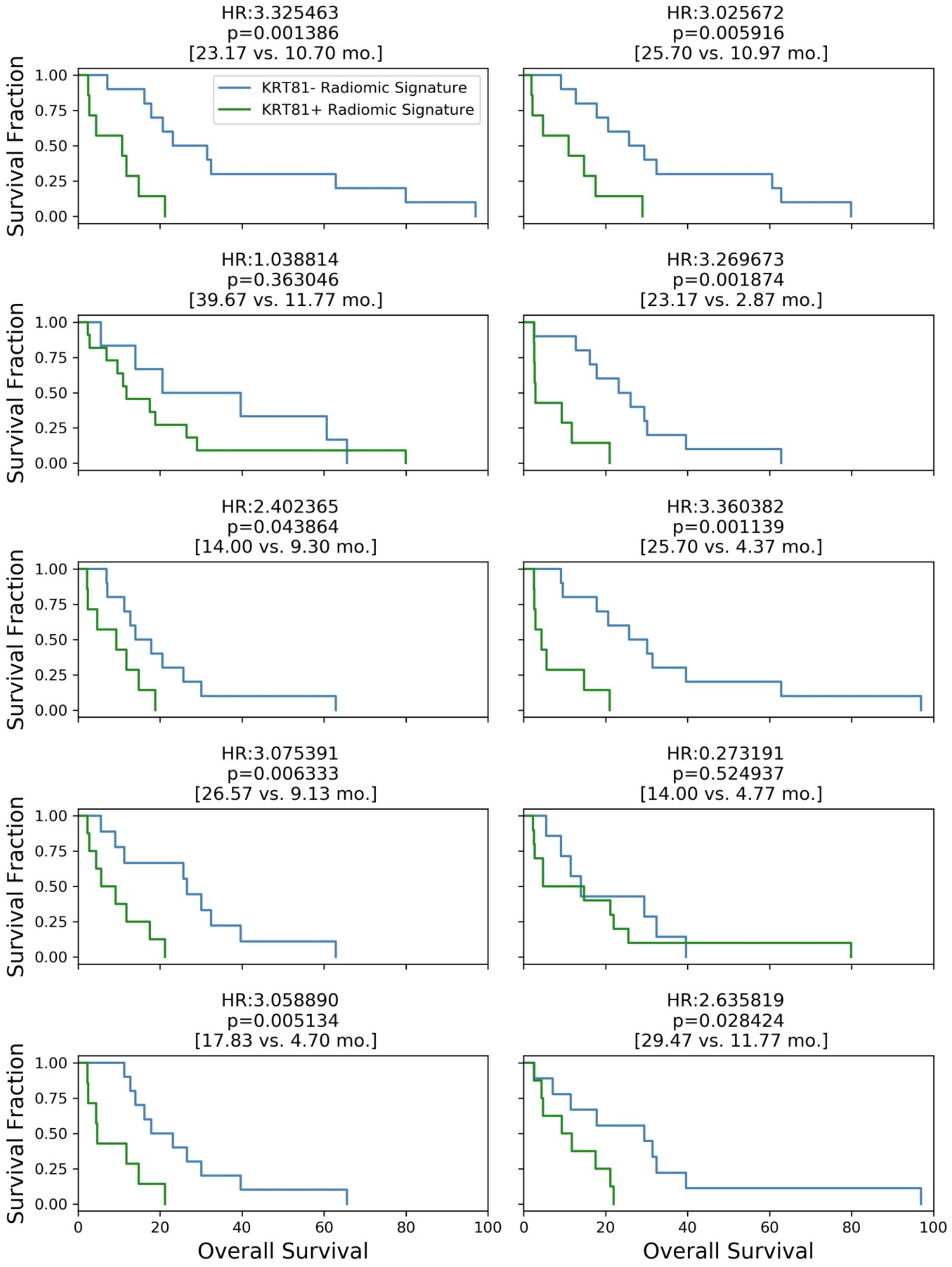

Kaplan-Meier curves resulting from 10-fold cross validation and showing overall survival stratified by radiomic signature. (KRT81+/*high risk* (blue line) or KRT81-/*low risk* (green line)). Total N=17.

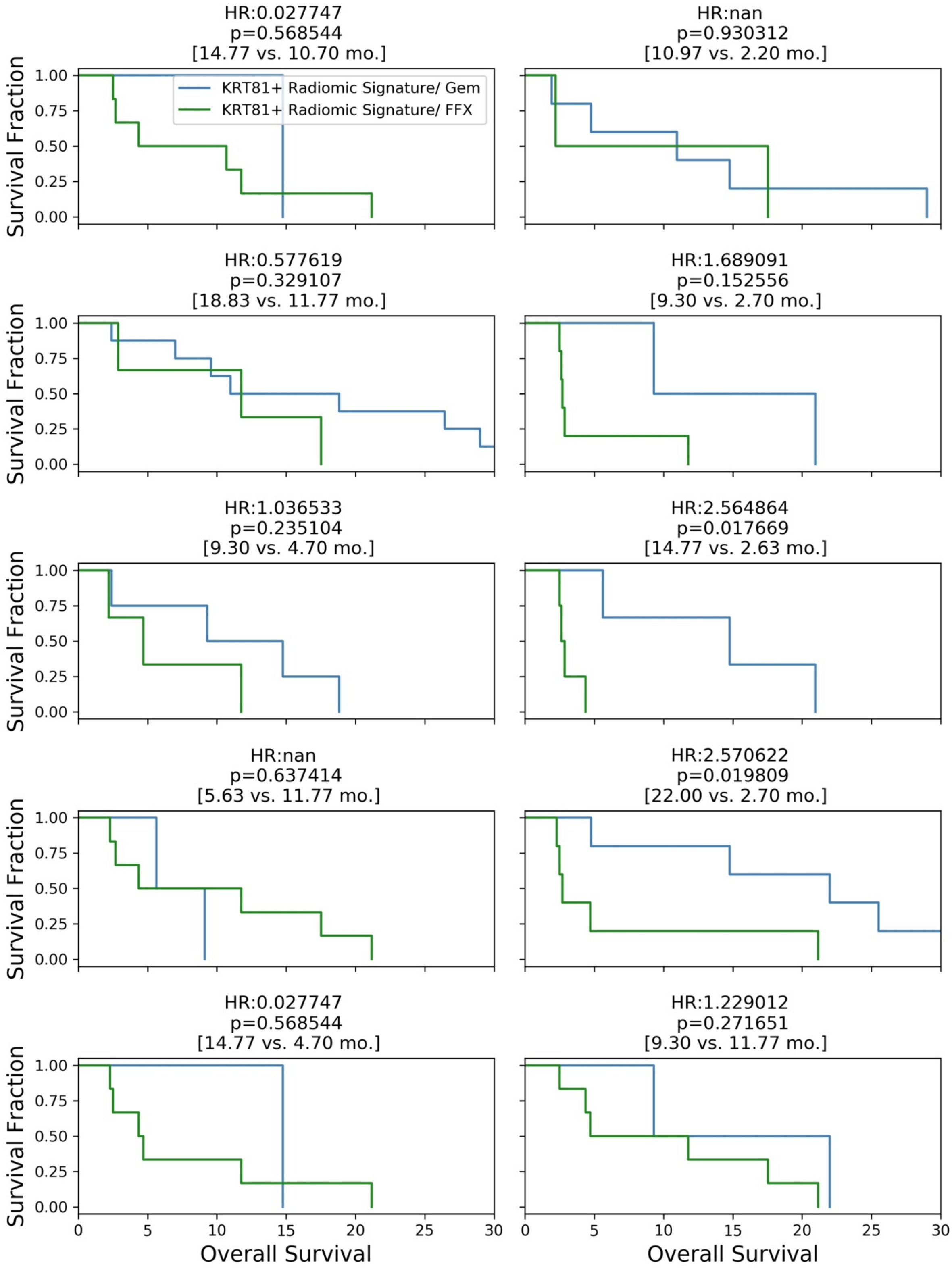

Kaplan-Meier curves resulting from 10-fold cross validation and showing overall survival stratified by radiomic signature and chemotherapy regimen. (KRT81+/*high risk* with gemcitabine (blue line) or FOLFIRINOX (green line)). Total N=7 to 11

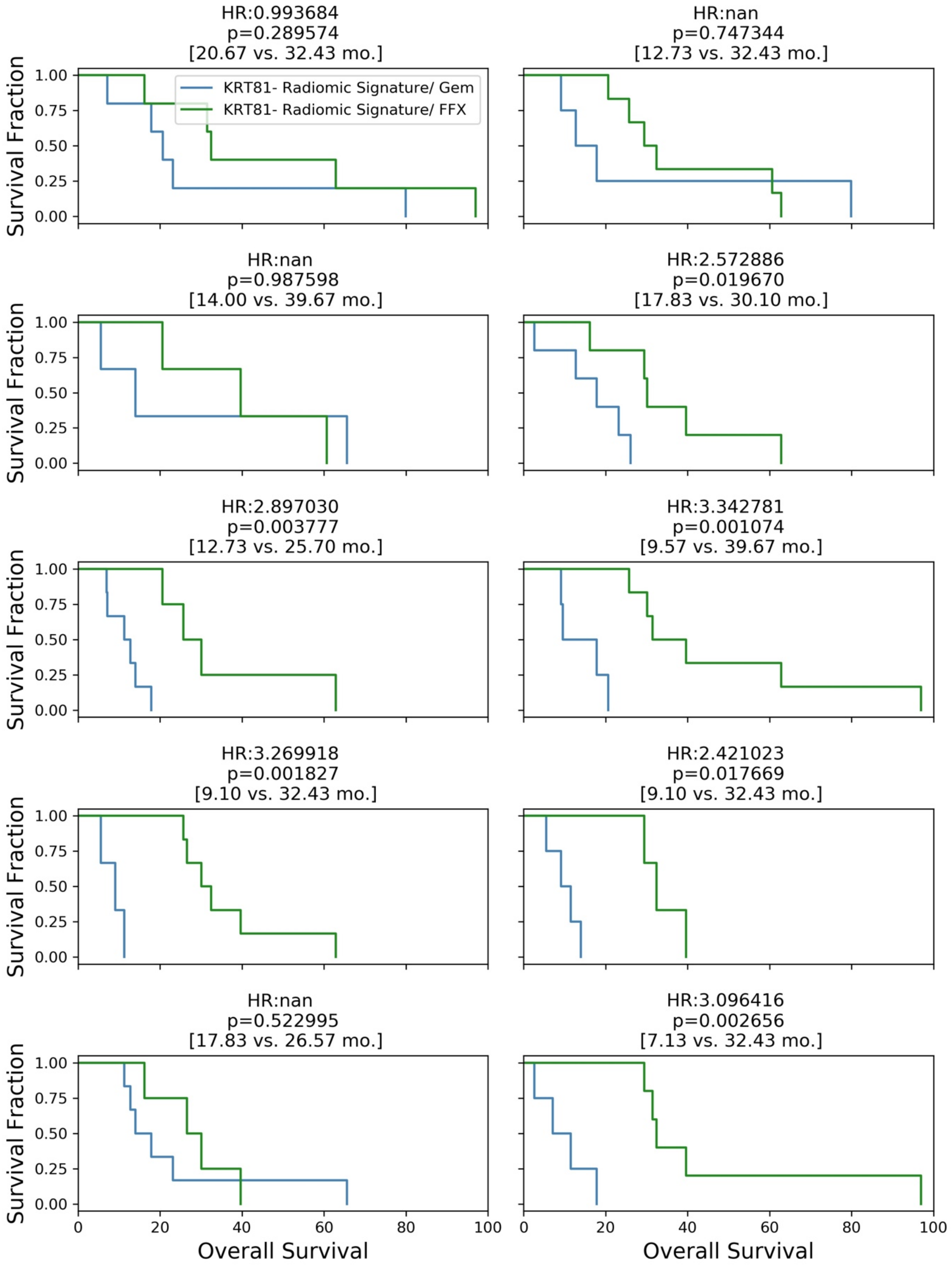

Kaplan-Meier curves resulting from 10-fold cross validation and showing overall survival stratified by radiomic signature and chemotherapy regimen. (KRT81-/*low risk* with gemcitabine (blue line) or FOLFIRINOX (green line)). Total N=6 to 10

7 STROBE Statement Checklist

	Item No	Reccomendation	Remark/ Location
**Title and abstract**	1	(*a*) Indicate the study's design with a commonly used term in the title or the abstract	Reported in abstract
		(*b*) Provide in the abstract an informative and balanced summary of what was done and what was found	Reported in abstract
**Introduction**			
Background/rationale	2	Explain the scientific background and rationale for the investigation being reported	Introduction
Objectives	3	State specific objectives, including any prespecified hypotheses	Introduction
**Methods**			
Study design	4	Present key elements of study design early in the paper	Mehods/ Study Design
Setting	5	Describe the setting, locations, and relevant dates, including periods of recruitment, exposure, follow-up, and data collection	Ibid.
Participants	6	(*a*) Give the eligibility criteria, and the sources and methods of selection of participants. Describe methods of follow-up	Ibid.
		(*b*) For matched studies, give matching criteria and number of exposed and unexposed	Ibid.
Variables	7	Clearly define all outcomes, exposures, predictors, potential confounders, and effect modifiers. Give diagnostic criteria, if applicable	Ibid. and Methods/ Clinical variables. Compare also Results
Data sources/ measurement	8*	For each variable of interest, give sources of data and details of methods of assessment (measurement). Describe comparability of assessment methods if there is more than one group	Methods/ Study Design
Bias	9	Describe any efforts to address potential sources of bias	Results
Study size	10	Explain how the study size was arrived at	Methods/ Study Design
Quantitative variables	11	Explain how quantitative variables were handled in the analyses. If applicable, describe which groupings were chosen and why	Results, Table 1
Statistical methods	12	(*a*) Describe all statistical methods, including those used to control for confounding	Methods
		(*b*) Describe any methods used to examine subgroups and interactions	Ibid.
		(*c*) Explain how missing data were addressed	Supplementary Material Results
		(*d*) If applicable, explain how loss to follow-up was addressed	N.A.
		(*e*) Describe any sensitivity analyses	Supplementary Material
**Results**			

Participants	13*	(a) Report numbers of individuals at each stage of study—eg numbers potentially eligible, examined for eligibility, confirmed eligible, included in the study, completing follow-up, and analysed	Methods/ Study Design Supplementary Material
		(b) Give reasons for non-participation at each stage	Ibid.
		(c) Consider use of a flow diagram	See below
Descriptive data	14*	(a) Give characteristics of study participants (eg demographic, clinical, social) and information on exposures and potential confounders	Methods/ Study design Results, Table 2
		(b) Indicate number of participants with missing data for each variable of interest	N.A.
		(c) Summarise follow-up time (eg, average and total amount)	Methods/ Study design
Outcome data	15*	Report numbers of outcome events or summary measures over time	Results
Main results	16	(*a*) Give unadjusted estimates and, if applicable, confounder-adjusted estimates and their precision (eg, 95% confidence interval). Make clear which confounders were adjusted for and why they were included	Results
		(*b*) Report category boundaries when continuous variables were categorized	N.A.
		(*c*) If relevant, consider translating estimates of relative risk into absolute risk for a meaningful time period	N.A.
Other analyses	17	Report other analyses done—eg analyses of subgroups and interactions, and sensitivity analyses	Results
**Discussion**			
Key results	18	Summarise key results with reference to study objectives	Discussion
Limitations	19	Discuss limitations of the study, taking into account sources of potential bias or imprecision. Discuss both direction and magnitude of any potential bias	Discussion
Interpretation	20	Give a cautious overall interpretation of results considering objectives, limitations, multiplicity of analyses, results from similar studies, and other relevant evidence	Discussion
Generalisability	21	Discuss the generalisability (external validity) of the study results	Introduction Discussion Supplementary Material
**Other information**			
Funding	22	Give the source of funding and the role of the funders for the present study and, if applicable, for the original study on which the present article is based	Preamble

The flowchart of patient recruitment is shown below:

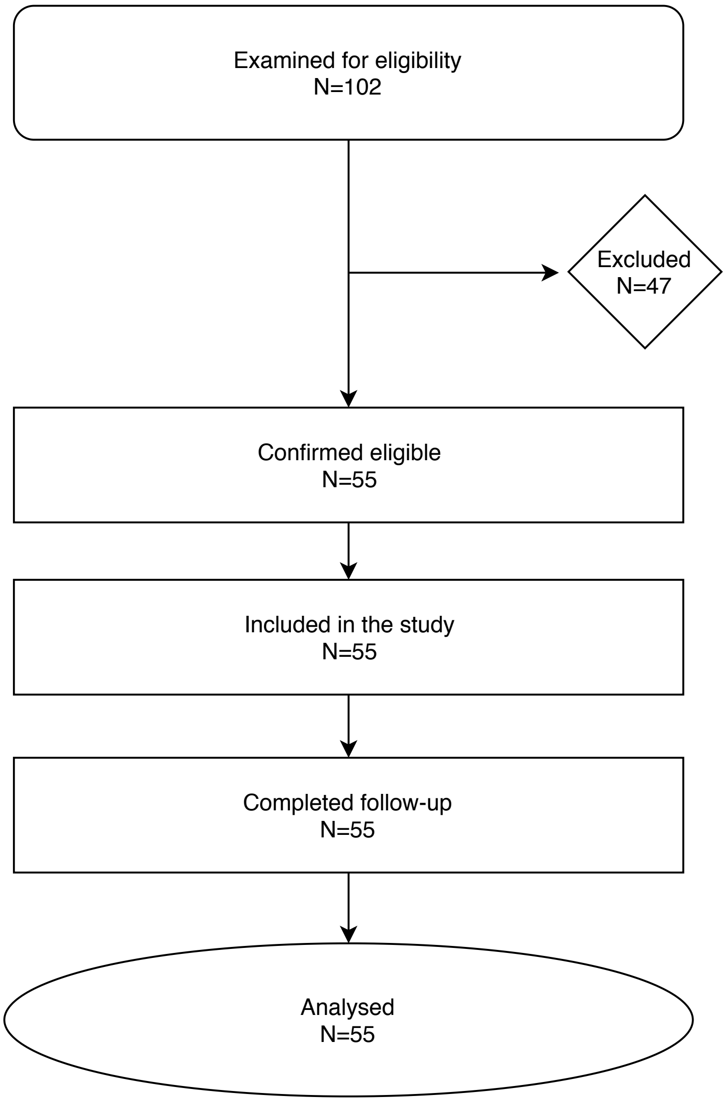

Reasons for exclusion:

No PDAC in final histopathological report (8 patients), insufficient or incomplete imaging (14 patients), death within 2 months of imaging (4 patients), received prior treatment (8 patients), histopathologically unclassifiable tumor subtype (13 patients)

8 Supplementary Bibliography

(1) Vallieres, C. R. Freeman, S. R. Skamene, and I. El Naqa. A radiomics model from joint FDG-PET and MRI texture features for the prediction of lung metastases in soft-tissue sarcomas of the extremities.Physics in medicine and biology, 60(14):5471–96, Jul 2015

(2) van Griethuysen, J. J. M., Fedorov, A., Parmar, C., Hosny, A., Aucoin, N., Narayan, V., Beets-Tan, R. G. H., Fillon-Robin, J. C., Pieper, S., Aerts, H. J. W. L. (2017). Computational Radiomics System to Decode the Radiographic Phenotype. Cancer Research, 77(21), e104–e107

Compare also: https://pyradiomics.readthedocs.io/

(3) PyRadiomics feature definitions:

https://pyradiomics.readthedocs.io/en/latest/features.html

Compare also: (4) below

(4) Zwanenburg, A., Leger, S., Vallières, M., and Löck, S. (2016). Image biomarker standardisation initiative: https://arxiv.org/abs/1612.07003v7

Multiple versions of the manual are available, version 7 is linked and was current at the time of the analysis

(5) McGraw, K. O., & Wong, S. P. (1996). Forming inferences about some intraclass correlation coefficients. *Psychological Methods, 1*(1), 30-46.

(6) Dancey C.P., Reidy J. Statistics for Psychology, Pearson Education; 2007
